# Hand-Book of Phthiscology

**Published:** 1889-06

**Authors:** 


Hand-book of Historical and Geographical Phthiscology, with special reference to the distribution of consumption in the United States; compiled and arranged by George A. Evans, M. D., Member of the Medical Society of the County of Kings, New York; member of the American Medical Association; formerly physician to the Atlantic Avenue and Bushwick, and East Brooklyn Dispensaries, etc., New York. D. Appleton & Co., 1888.

This work, which contains a historical sketch of phthisis, from the time of Hippocrates down to the present time is in a large degree a translation of the interesting and valuable German work of "Die Luberculose," etc., by Waldmberg, which was published in Berlin in 1869.

It presents in a clear and able way much interesting matter treated under the following general divisions:

1. Historical Sketch

2. Geographical distribution of consumption in countries other than the United States.

3 Geographical distribution in the U. S.

4 Topography and climate of states and territories, and summary for states, groups, cities, and for counties of ten thousand population and upward; showing the number of deaths from consumption per one thousand inhabitants.

5. Meteorology.

6. Etiology.

7. Conclusions.

